# Nonlinear Creep Amplification Factor Considering Damage Evolution of Concrete under Compression

**DOI:** 10.3390/ma15196742

**Published:** 2022-09-28

**Authors:** Zuanfeng Pan, Dong Cao, Bin Zeng, Yuwei Wang

**Affiliations:** 1 State Key Lab of Disaster Reduction in Civil Engineering, Tongji University, Shanghai 200092, China; 2 Department of Structural Engineering, Tongji University, Shanghai 200092, China; 3 Central Research Institute of Building and Construction of MCC Group, Beijing 100088, China

**Keywords:** concrete, nonlinear creep, stress level, damage, amplification factor

## Abstract

Creep affects the long-term deformation of concrete structures. Nonlinear creep further overestimates the safety factor of structures and affects the safety service performance. The coupling of creep and a damage model considering the rate effect is conducive to accurate prediction of nonlinear creep, but the iterative process of strain makes the calculation method more complex. The purpose of this study is to propose a nonlinear creep explicit method that considers the damage evolution of concrete under compression. Two groups of axial compression members with compressive stresses of 0.2 fc and 0.4 fc were made. Considering the law of concrete damage evolution under uniaxial compression, coupled with elastic creep and damage incremental strain, the lower limit of the medium stress level that gives rise to nonlinear creep is analyzed. The concrete nonlinear creep amplification coefficient with a loading age of 28 days and loading duration of 360 days is studied with consideration for the uncertainty of relative humidity and the theoretical thickness of the component. On this basis, the explicit calculation formula of the nonlinear creep amplification coefficient related to the concrete axial compressive strength and stress level is given. The results indicate that the nonlinear creep amplification coefficient increases nonlinearly with an increase in the stress level, and, when the compressive stress level ratio is higher than 0.6, the nonlinear creep amplification coefficient increases significantly; when the stress level is determined, the creep amplification coefficient decreases gradually with an increase in the compressive strength of the concrete. It is suggested that a stress level range of 0.35~0.75 should be used for the study of a nonlinear creep amplification factor under the medium stress state.

## 1. Introduction

Creep has an important influence on the long-term behavior of concrete structures. Creep refers to the phenomenon in which a concrete structure is continuously compressed, and the strain deformation increases over time. Generally, creep refers to linear creep. A nonlinear creep phenomenon is mainly caused by micro-cracks appearing at the interface between the aggregate, solidified cement slurry and internal mortar [1,2]. Nonlinear creep behavior may exist due to concrete cracking and local high-pressure stress. Crack extension damage due to concrete cracking has received much attention [3,4]. Dénarié [5] argued that crack extension and creep effects affect the long-term deformation properties of concrete. Challamel [6] developed a time-dependent model suitable for concrete brittle materials that considers the possible interaction between concrete strength softening and cracking. For the delayed effect of concrete cracking, Torrenti et al. [7] analyzed the coupling of shrinkage, creep and cracking from a hydrodynamic point of view. Rossi [8] provided an explanation for the delayed effect of concrete under continuous loading. It was shown that the evolution of concrete cracking causes the delayed behavior of concrete, which eventually results in the extension of macroscopic cracks on the concrete surface. Yu et al. [9] investigated the relationship between nonlinear creep, damage and cracking in concrete based on the discrete element theory. It was found that concrete microcracks first appeared at the interface, where the cement gel in the cement mortar showed stress redistribution after viscous flow and produced new microcracks, which then expanded further, eventually leading to concrete cracking and damage.

It is well known that creep is potentially harmful to the safety, durability and service properties of concrete structures. The appearance of nonlinear creep further overestimates the structural safety factor, which seriously affects the overall performance and public safety of the structure. The creep coefficient is used to describe the concrete creep response, considering the environmental temperature, relative humidity, concrete age and other factors related to concrete [10,11,12,13,14,15]. A large amount of research data shows that concrete creep under compression follows the laws: at a low stress level, the creep rate decreases gradually with time, and the creep develops linearly over time with no prominent material damage; at a medium stress level, nonlinear creep occurs; at a high stress level, the concrete creep deformation rate increases continuously, and the concrete structure degrades gradually.

In common practice, linear creep of concrete is considered to produce deformations that increase over time; however, through an ultimate limit state analysis, the compressive stress level can be moderately high when designing the concrete structural members [16]. According to the elastic creep theory, the creep strain and stress of concrete develop linearly, but, under the conditions of medium stress and high stress, the concrete creep strain is much larger than the concrete creep calculated from the elastic stress–strain relationship of concrete, mainly because the damage caused by concrete compression is ignored, including the development of interface cracks and internal mortar cracks. The development of concrete cracks is divided into three stages [17]: In the first stage, the concrete cracks develop slowly and show linear creep. With an increase in the stress level (stress level *η* = 0.4 ~ 0.8), the concrete interface cracks grow rapidly, and the creep is nonlinear. In the third stage, the concrete internal mortar cracks and interface cracks develop rapidly until the concrete structure fails. A group of circular concrete structures was tested by Mazzotti et al. [18] under cyclic loading to study the change in Poisson’s ratio of concrete creep, and the nonlinear regression curve of the creep damage index was obtained. Sellier [19] simulates nonlinear creep, multiaxial creep and dry creep within the framework of pore mechanics. It is found that the nonlinear dependence of creep on a strong load can be based on the criterion of using equivalent shear stress.

At present, the use of damage mechanics to study concrete creep is the method chosen by the majority of scholars [20]. The following two types of methods exist for predicting nonlinear creep characteristics by coupling creep effects to concrete subjected to compression damage: In the first type of method, as the concrete creep strain calculated by the elastic creep theory is smaller than the real strain of the structure, in order to simplify the calculation method, some scholars have proposed the simulation of nonlinear creep by multiplying the elastic creep strain by an amplification factor [13,14,18,21,22,23]. An experimental study on concrete creep under medium stress was carried out by Tang [21]. The weighted average value of the linear creep amplification coefficient at different load holding time points of each stress level in the test group is taken as the nonlinear creep coefficient, and the linear creep model and the convergent nonlinear creep model are unified. In reference to the rising section of the concrete stress–strain curve, a formula for calculating the nonlinear creep amplification coefficient was given by Ruiz et al. [23] through the extension of the aging coefficient method. In the second type of method, Bažant et al. [24] assumed that the bond between molecules of concrete under compression is proportional to the loading rate of the load and argued that the development of cracks within the concrete would expand at a specific rate with the load. A number of novel creep models have been developed by researchers based on the B3 and B4 models [25,26,27]. Dummer [25] proposed an extended concrete damage plasticity material model that considers damage evolution. Yu [26] developed a coupled nonlinear creep and damage model that considers the rate effects at moderate to high loading levels. Characterized by the creep of concrete behavior, it was developed based on the B3 model, while the rate effects were achieved by modifying the damage model through a strength amplification factor. Bažant [28] proposes an explicit algorithm for a step-by-step finite element analysis that considers the cracking rate dependence and short-time creep. A good approximation of the rate effects observed in concrete experiments is obtained.

The mechanism of concrete nonlinear creep under compression is complex. In addition to the studies based on elastic creep, theoretical research on concrete nonlinear creep exists, mainly including the viscoelastic plastic theory, elastic–plastic theory and nonlinear elastic creep constitutive model [29]. In recent years, some progress has been made regarding the research of concrete nonlinear creep under a medium compressive stress level with the adoption of the more accurate but more complex rheological model [16,30,31,32]. In this study, based on the damage evolution law of concrete under compression, the nonlinear creep amplification coefficient is studied by considering multi-level factors, such as damage, concrete strength and stress level. A nonlinear coupling model of concrete creep under different compressive stress levels is constructed by coupling the damage incremental strain with the elastic creep, and the damage incremental strain is caused by the concrete damage evolution under compression. The interval variable of the creep amplification coefficient under relative conditions is given.

## 2. Medium Stress Level

### 2.1. Experimental Preparation

It is generally accepted that the stress level that produces nonlinear creep is within the range from 0.4 to 0.8 and that macro-brittle damage will occur in a short time if the compressive stress of the concrete exceeds 0.8*f*_c_ [20]. Hamnd [33] argues that stress levels greater than 0.3 reflect the characteristics of nonlinear creep in concrete, not only is the instantaneous strain nonlinear, but the creep is also nonlinear. Yu [9] argues that when the stress strength is greater than 0.5, there is a nonlinear relationship between the creep deformation and stress level, known as nonlinear creep. It can be seen that there is no consensus on the limit of stress that produces nonlinear creep. Smadi [34] tested the creep of low, medium and high compressive strength concrete cylinders at a stress level of 0.4–0.8. The study found that the creep strain and creep coefficient of high-strength concrete were smaller than those of low-strength and medium-strength concrete. The creep of high-strength concrete maintains a linear relationship when the stress level is 0.65, and the creep of the medium- and low-strength concretes still shows a linear relationship when the stress level is 0.45. It can be seen that nonlinear creep may also be related to the compressive strength of concrete. Moradi [35] developed a convenient method based on machine learning to predict the compressive strength of concrete at different ages. The critical value between linear creep and nonlinear creep has been studied by Tang [21]. Through a comparative analysis of the nonlinear creep test data and linear creep test data, the stress level of the nonlinear creep of concrete is determined to be in the range from 0.35*f*_c_ to 0.76*f*_c_. Through four groups of prism tests with different load ratios *η* = 0.32, 0.48, 0.64 and 0.8, conducted by Hu and Gu [36], it was determined that the stress level limit between the nonlinear creep and linear creep of concrete is between 0.32*f*_c_ and 0.48*f*_c_.

**Figure 1:**
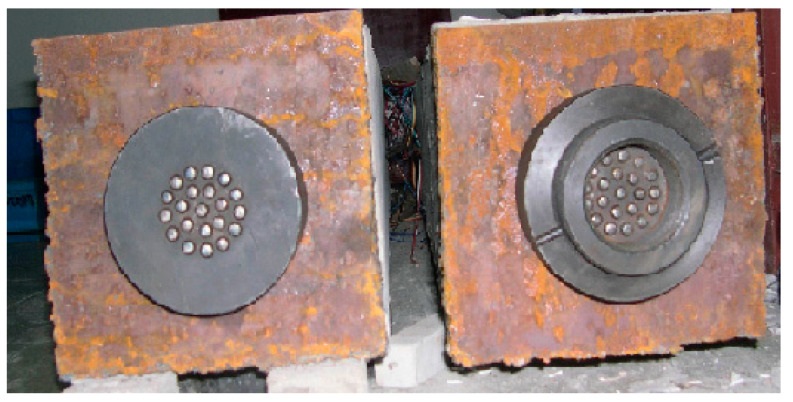
Prestressed concrete “series connected” creep specimens.

In order to study the lower limit of the stress level when nonlinear creep initiates, two groups of prestressed concrete specimens are designed, and the creep strain development law of prestressed structure under a low stress level is analyzed. The concrete strength grade is 30 MPa; the size of the specimen is 250 mm × 250 mm × 2000 mm; the compressive stress ratios of the concretes equal 0.2*f*_c_ and 0.4*f*_c,_ respectively; and there is a hole in the specimen section to allow the post-tensioned tendons through. A total of 21 high-strength steel wires with a diameter of 7 mm are tensioned to apply a compressive force on the specimens. Details about the two anchoring locations of the component are shown in Figure 1. When the prestress loss is more than 3% of the initial prestress force, a compensating tension is provided. Considering the combined effects of concrete shrinkage, creep and tendon relaxation, the creep coefficient of plain concrete under constant stress in the test specimens can be reversed.

After being cast, the specimens were stored in a moist room where the ambient temperature was 20 ± 2 °C, and the relative humidity was approximately 95%. After standardly curing for 14 days, they were dried in a constant temperature (20 ± 2 °C) and constant humidity (60 ± 5%) room for loading, and the environmental conditions were maintained throughout the test. The stress levels correspond to 0.2 and 0.4 times the axial compressive strength of the concrete at the same time. The shrinkage strains of the two groups of specimens under the same environment were measured simultaneously.

The creep coefficient refers to the ratio of creep deformation to the instantaneous elasticity under loading, and the calculation method is as follows:

(1) $$\phi (t,t_{0})=\frac{\varepsilon_{c}(t,t_{0})}{\varepsilon_{e}(t_{0})}=\frac{\varepsilon_{\mathrm{total}}(t,t_{0})-\varepsilon_{e}(t_{0})-\varepsilon_{\mathrm{sh}}(t,t_{0})}{\varepsilon \left(t_{0}\right)}$$

where *ε*_total_(*t*, *t*_0_) is the total strain of the concrete from the loading time *t*_0_ to the time *t*; *ε*_e_(*t*, *t*_0_) is the instantaneous elastic strain of the concrete under loading; *ε*_sh_(*t*, *t*_0_) is the shrinkage strain of the parallel specimen from *t*_0_ to *t*.

Table 1 shows the measured values of the compressive strength and modulus of elasticity of the members at different ages. The dimension of the cube is 150 mm × 150 mm × 150 mm, and the prism is 150 mm × 150 mm × 300 mm. After standardly curing for 14 days, the specimens were dried in 60% relative humidity. The concrete age at loading, *τ*, of the two specimens is 14 days. In each specimen, one vibrating wire strain sensor produced by Haiyan Engineering Material Instrument Co., Ltd., China, was buried in the middle of each segment longitudinally. On the surface of specimens, the brass inserts were cast into each specimen, such that two gauge points separated by 250 mm could be attached after curing. The YB-250 handheld strainmeter produced by Tianjin Construction Instruments Co., Ltd. was used for measuring the shrinkage strains. Because the values measured by the strain sensor and two brass inserts are relatively close, the former values are regarded as the strains of the specimens.

### 2.2. Test Results

**Figure 2:**
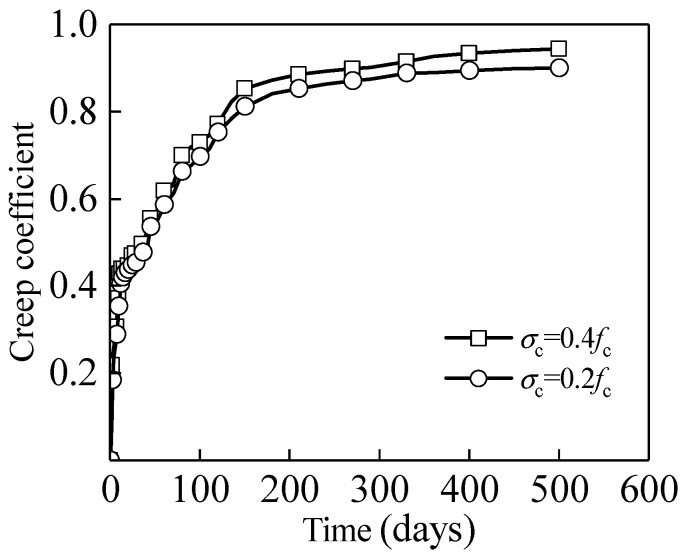
Variation curve of concrete creep coefficient.

In order to facilitate the analysis, the measured deformation of a specimen is transformed into the creep coefficient. The results are shown in Figure 2. The creep coefficient trend of specimens under the action of 0.2*f*_c_ and 0.4*f*_c_ stress levels is close, which is in line with the rapid development of creep in a short period of time, and the deformation rate decreases after a certain period of time. The creep of a concrete specimen develops rapidly within the first 180 days of bearing load. It can be seen from the figure that the creep coefficient of the concrete specimen reaches 0.841 and 0.872 under the action of 0.2*f*_c_ and 0.4*f*_c_ stress levels, respectively. After 180 days, the creep strain development slows down gradually. At 500 days, the creep coefficients are 0.900 and 0.944, respectively.

This experiment is carried out on test specimens with the same concrete design strength, loading age, applied post-tension force and environmental conditions. According to the elastic creep theory, the concrete creep coefficient should be the same under the action of a low stress level. It can be seen from Figure 2 that the creep coefficient test results are different from the linear creep theory, which may be due to some slight damage to the concrete at the moment of loading under the low stress level of 0.4*f*_c_. Because the damage is less significant, the creep coefficient difference is small. Considering the damage accumulation and the complexity of the concrete component’s service environment, the concrete damage under 0.4*f*_c_ compressive stress should be considered. It is worth noting that this key point is less considered in the previous studies of other scholars. Generally, the upper limit of the stress level for the medium stress level is 0.8*f*_c_. The literature [21] fits the existing test data and finds that, when the stress level is close to 0.8*f*_c_, the concrete structure has already experienced rapid failure. The linear creep theory does not apply at this stress level, so the nonlinear creep theory was developed, as mentioned previously. The nonlinear creep of the concrete structure under a stress level higher than 0.76 is affected by the concrete strength, loading mode and other factors. In addition, it is seldom encountered in actual applications. Therefore, the limit of the nonlinear creep under the medium stress level is considered to be 0.35*f*_c_~0.75*f*_c_ in this paper. Under high compressive stress, the concrete members will be destroyed in a short time. The nonlinear creep in this stage is temporarily not considered here; only the development law of nonlinear creep in the medium stress level is studied.

## 3. Nonlinear Creep Amplification Factor Considering Damage Evolution

### 3.1. Calculation Method of Linear Creep

At present, the linear creep calculation method of concrete is mainly based on the elastic creep theory, and the detailed creep coefficient calculation models are given in the codes, ACI209 [1], EC2 [2], CEB-FIP [10] and GB50010 [15], respectively. According to the superposition principle, the creep strain of concrete under compression is as follows:

(2) $$\varepsilon (t)=\sigma (\tau_{0})[\frac{1}{E(\tau_{0})}+C(t,\tau_{0})]+{ʃ}_{\tau_{0}}^{t}\left[\frac{1}{E(\tau_{0})}+C(t,\tau_{0})]d\sigma (\tau \right)$$

in which *C*(*t*, *τ*_0_) is the creep degree, and *τ*_0_ is the initial loading age. Performing a divisional integration on the above equation [13,37]:

(3) $$\varepsilon (t)=\frac{\sigma (t)}{E(t)}-{ʃ}_{\tau_{0}}^{t}\frac{\partial }{\partial \tau }[\frac{1}{E(\tau )}+C(t,\tau )]\sigma (\tau )d\tau$$

Equation (3) is the stress–strain relationship based on the elastic creep theory, but the above equation contains the integral of the entire stress history, which is inconvenient for the practical application of engineering calculations. Apply the median integral theorem to the creep constitutive equation of concrete under compression [38]:

(4) $$\varepsilon (t)=\frac{\sigma (\tau_{0})}{E(\tau_{0})}[1+\varphi (t,\tau_{0})]+\left[\sigma (t)-\sigma (\tau_{0})\right]/E(t,\tau_{0})$$

(5) $$E(t,\tau_{0})=E(\tau_{0})/\left[1+\chi (t,\tau_{0})\varphi (t,\tau_{0})\right]$$

where *E*(*t*, *τ*_0_) is the effective modulus, *φ*(*t*, *τ*_0_) is the creep coefficient and *χ*(*t*, *τ*_0_) is the aging coefficient:

(6) $$\chi (t,\tau_{0})=\frac{1}{1-R(t,\tau_{0})}-\frac{1}{1-\varphi (t,\tau_{0})}$$

The relaxation coefficient is taken as *R*(*t*, *τ*_0_) = 0.91*e*^−0.686*φ*(*t*, *τ*0)^ for the elastic continuation and plastic flow theory and as *R*(*t*, *τ*_0_) = *e*^−*φ*(*t*, *τ*0)^ for the aging theory [37]. If the duration of loading is longer, the aging coefficient is approximately 0.82.

According to the elastic creep theory, the total strain of concrete can be expressed as follows:

(7) $$\varepsilon_{c}(t,\tau_{0})=\varepsilon_{\mathrm{ce}}(t,\tau_{0})+\varepsilon_{\mathrm{cr}}(t,\tau_{0})$$

where *ε*_ce_(*t*, *τ*_0_) is the elastic strain, and *ε*_cr_(*t*, *τ*_0_) is the creep strain.

**Figure 3:**
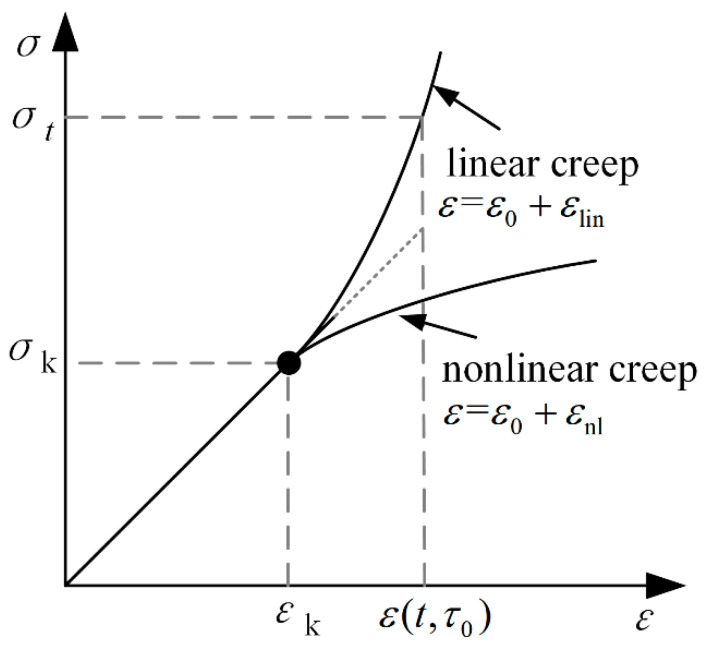
Creep development curve of concrete with stress.

Under a lower compressive stress level, the elastic creep theory is better able to respond to the creep strain of concrete [39], but, under a moderate or high stress level, the concrete will generate nonlinear creep under compression. Equation (7) underestimates the concrete creep strain under the medium stress state [16,40]. As shown in Figure 3, when the stress level is less than the lower limit of the nonlinear stress level *σ_k_*, the elastic creep theory is in good agreement with the measured values, but, as the compressive stress increases, the stress–strain curve gradually appears nonlinear, and the concrete deformation rate continues to increase. Therefore, when the concrete compressive stress is higher than the lower limit of the nonlinear creep stress level, the influence of nonlinear creep should be considered.

### 3.2. Calculation Method of Nonlinear Creep Considering Damage

It is assumed that the damage will accumulate gradually in the process of concrete compression. When the maximum damage is reached, the damage will not continue to increase. The damage increment is defined according to the creep strain of concrete that develops with the duration. Considering the influence of damage evolution on concrete creep under compression, coupled with the damage strain produced by the concrete damage evolution, the concrete total strain under compression is defined as follows:

(8) $$\varepsilon_{c}(t,\tau_{0})=\varepsilon_{\mathrm{ce}}(t,\tau_{0})+\varepsilon_{\mathrm{cr}}(t,\tau_{0})+\varepsilon_{d}(t,\tau_{0})$$

In Equation (8), $\varepsilon_{d}(t,\tau_{0})$ is the incremental strain, which considers the compressive damage evolution of concrete.

According to the continuum damage mechanics, using the isotropic continuum damage formula, the constitutive relationship of the instantaneous load is as follows [16]:

(9) $$\{\begin{array}{l} \varepsilon_{ij}^{\mathrm{el},d}(t,\tau_{0})=\frac{1+\upsilon }{E_{0}(1-d)}\sigma_{ij}-\frac{\upsilon }{E_{0}(1-d)}\sigma_{kk}\delta_{ij}\\ \varepsilon_{ij}^{\mathrm{el},d}(t,\tau_{0})=\varepsilon_{ij}^{\mathrm{el}}(t,\tau_{0})+\varepsilon_{ij}^{d}(t,\tau_{0}) \end{array}$$

in which $\varepsilon_{ij}^{\mathrm{el},d}$ represents the damage elastic strain, the unidirectional compressive stress tensor of concrete *σ*_22_ = *σ*_33_ = 0, *E*_0_ is the initial elastic modulus of concrete, *δ_ij_* is the Dirichlet function and *d* is the damage degree of the concrete during compression creep. Expressing the transverse strain as a function of the longitudinal strain, the longitudinal strain–stress relationship of the concrete is expressed as follows:

(10) $$\varepsilon_{ij}^{\mathrm{el},d}(t,\tau_{0})=\varepsilon_{ij}^{\mathrm{el}}(t,\tau_{0})+\varepsilon_{ij}^{d}(t,\tau_{0})=\frac{\sigma_{ij}}{E_{0}(1-d)}$$

Therefore, the coupled concrete damage evolution and creep should become:

(11) $$\varepsilon_{\mathrm{cr},d}(t,\tau_{0})=\varepsilon_{\mathrm{cr}}(t,\tau_{0})+\varepsilon_{d}(t,\tau_{0})=\frac{\sigma \varphi }{E_{0}(1-d)}$$

where *φ* is the creep coefficient. Then, at the time *t*, the creep strain increment caused by the compression damage evolution of concrete can be written as:

(12) $$\Delta \varepsilon_{d}(t,\tau_{0})=\frac{\sigma }{1-d(t)}[c_{d}(t,\tau_{0})-c(t,\tau_{0})]$$

in which *c*_d_(*t*, *τ*_0_) is the creep degree, which considers the compression damage evolution of the concrete, and *c*(*t*, *τ*_0_) does not consider the concrete damage. The damage evolution parameter *d* can be calculated as follows [15]:

(13) $$d=1-\frac{n\rho }{n-1+x^{n}}$$

(14) $$x=\frac{\varepsilon_{\mathrm{cr}}(t,\tau_{0})}{\varepsilon_{r}}$$

where *ρ* = *f*_cr_/(*Eε*_r_), *n* = *Eε*_cr_/(*Eε*_r_ − *f*_cr_) and *f*_cr_ is the representative value of the uniaxial compressive strength of concrete. *f*_cr_ can be taken as the prismatic compressive strength *f*_ck_, the cube compressive strength *f*_cu_ or the average compressive strength *f*_cm_. *ε*_r_ is the peak compressive strain of the concrete, and $\varepsilon_{r}=(700+172\sqrt{f_{\mathrm{ck}}})\times 10^{-6}$.

**Figure 4:**
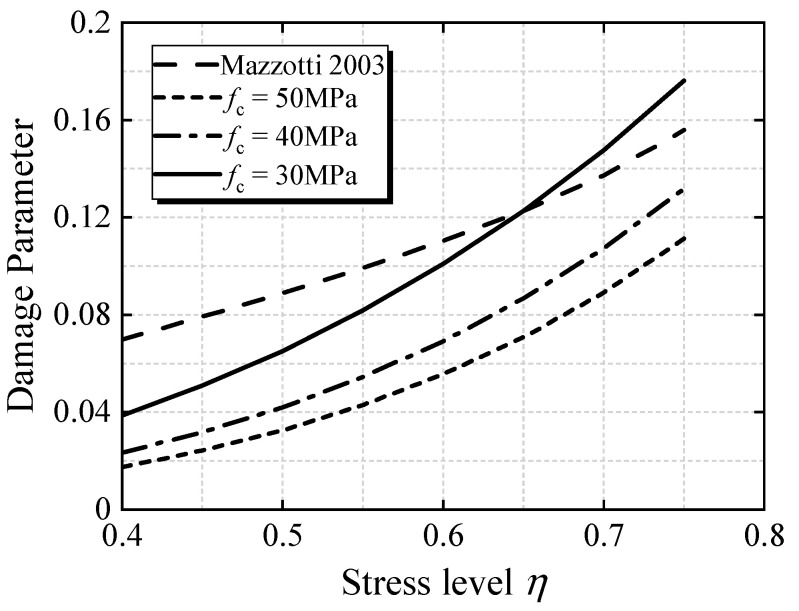
Variation curve of damage parameters with stress level, compared with Mazzotti [16].

The change trend of the damage parameters with the stress level is plotted in Figure 4, and, compared with the regression curve of the damage index in reference [18], it can be seen that the damage evolution parameters are obviously different when the concrete strength varies. In reference [18], the damage parameters do not consider the influence of the change in concrete strength. The lower the compressive strength of cylinder *f*_c_ is, the greater the concrete component damage is. With the increase in the stress level, the damage evolution gradually increases, which is consistent with the phenomenon that the creep rate of concrete increases significantly at higher stress levels. When the stress level is less than 0.65, the damage parameter value obtained from the literature [18] is higher than that of the *f*_c_ = 30 MPa curve. When the stress level is less than 0.65, the change in the damage parameter is higher than that of the *f*_c_ = 30 MPa curve. It can be seen that selection of the damage parameter has a great influence on the calculation of nonlinear creep.

**Figure 5:**
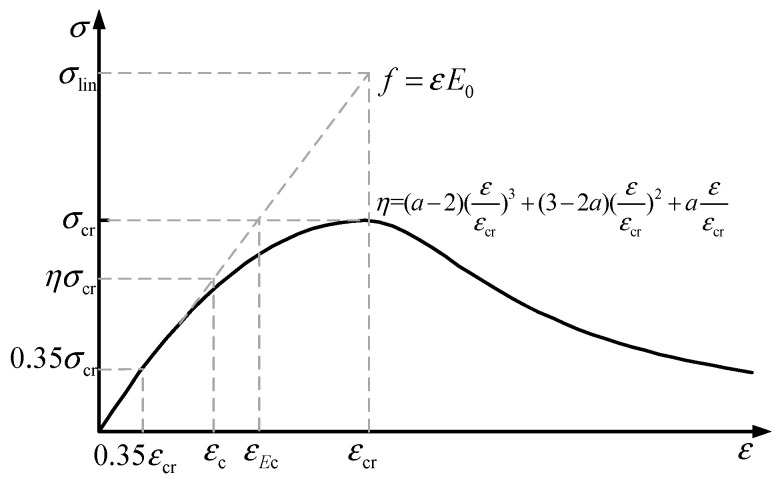
Stress–strain curve of concrete.

The stress–strain curve of concrete under uniaxial compression is shown in Figure 5. It can be seen that, if the concrete elastic modulus does not change (i.e., no changes in concrete rigidity), the stress–strain curve develops linearly along a straight line. The maximum stress *σ*_lin_ = *E*_0_*ε* can be reached, and the maximum stress considering the damage evolution is only *σ*_cr_. Creep is considered to develop linearly at a stress level of 0.35*σ*_cr_.

Since the descending section of the concrete compressive stress–strain curve represents the residual strength of the concrete, this article analyzes the rising section of the compressive stress–strain curve, and the curve equation of the rising section is [15,41]:

(15) $$\eta =(a-2){\left(\frac{\varepsilon }{\varepsilon_{\mathrm{cr}}}\right)}^{3}+(3-2a){\left(\frac{\varepsilon }{\varepsilon_{\mathrm{cr}}}\right)}^{2}+a(\frac{\varepsilon }{\varepsilon_{\mathrm{cr}}})$$

where *η* represents the compressive stress level of the concrete, and the value of *a* is in the closed interval [1.5, 3]. For the sake of simplicity, this paper uses *a* = 2. Under a constant stress level, the stress level *η* is a fixed value. Therefore, the damage of the concrete is expressed as:

(16) $$d_{e}=1-\frac{n\rho }{n-1+{\left(1-\sqrt{1-\eta }\right)}^{n}}$$

When the damage evolution parameter *d*(*τ*) = *d*_e_ of the concrete has completed damage accumulation, the incremental creep strain is:

(17) $$\varepsilon_{\mathrm{cd}}(t,\tau_{0})=\sum_{\Delta t}^{\tau }\Delta \varepsilon_{d}(t,\tau_{0})$$

in which *τ* is the cumulative completion time of the concrete damage, and Δ*t* is the incremental damage evolution time. Substituting the above formula into Equation (4), which considers the creep calculation method for the damage evolution of concrete during compression yielding:

(18) $$\varepsilon (t,\tau_{0})=\frac{\sigma (\tau_{0})}{E(\tau_{0})}[1+\varphi (t,\tau_{0})]+\sum_{\Delta t}^{\tau }\Delta \varepsilon_{d}(t,\tau_{0})+\left[\sigma (t)-\sigma (\tau_{0})\right]/E(t,\tau_{0})$$

Define the creep amplification factor as the ratio of the incremental strain to the elastic creep strain of the damage evolution of concrete:

(19) $$A_{v}=\frac{\varepsilon_{\mathrm{cr}}(t,\tau_{0})+\varepsilon_{\mathrm{cd}}(t,\tau_{0})}{\varepsilon_{\mathrm{cr}}(t,\tau_{0})}$$

Therefore, the creep strain calculations in Equations (8) and (18) under a medium stress level can be simplified as follows:

(20) $$\varepsilon_{c}(t,\tau_{0})=\varepsilon_{\mathrm{ce}}(t,\tau_{0})+\varepsilon_{\mathrm{cr}}(t,\tau_{0})A_{v}$$

Based on the elastic creep theory, the above formula considers the damage evolution of concrete, and the creep coefficient in the code can still be used. Compared with the incremental representation method of the rheological model, Equation (18) simplifies the calculation of nonlinear creep.

## 4. Amplification Factor

### 4.1. Formula Fitting

**Figure 6:**
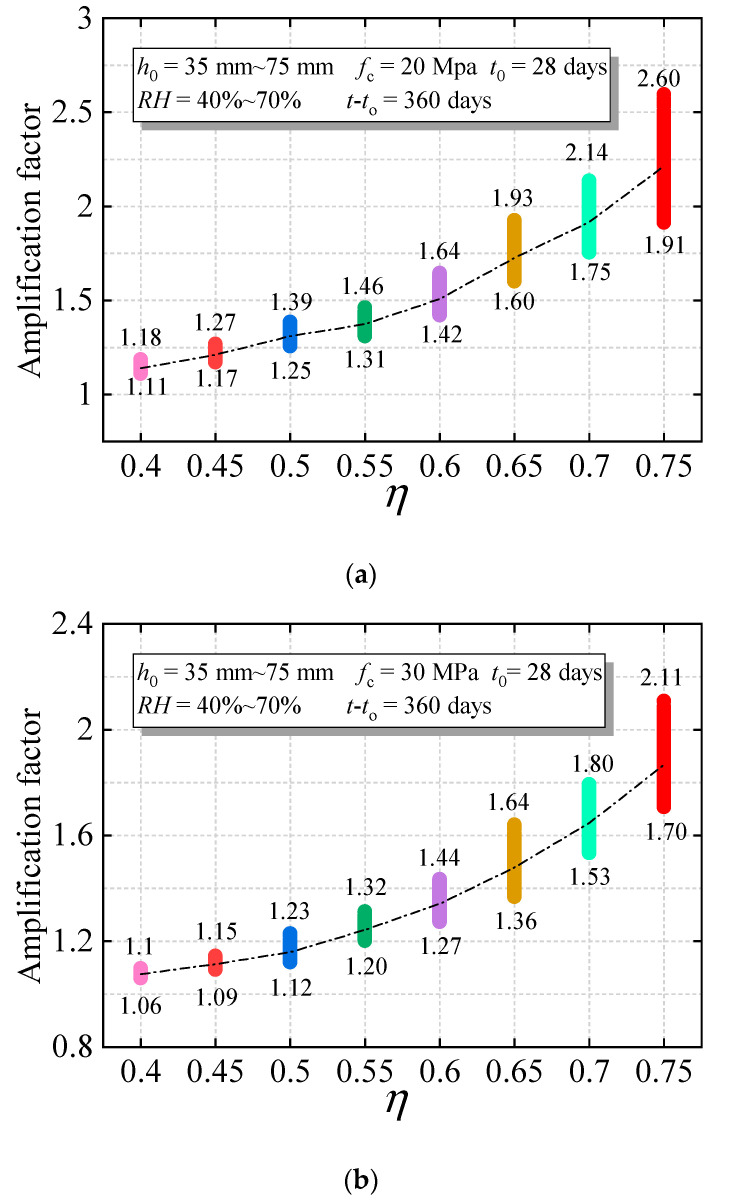

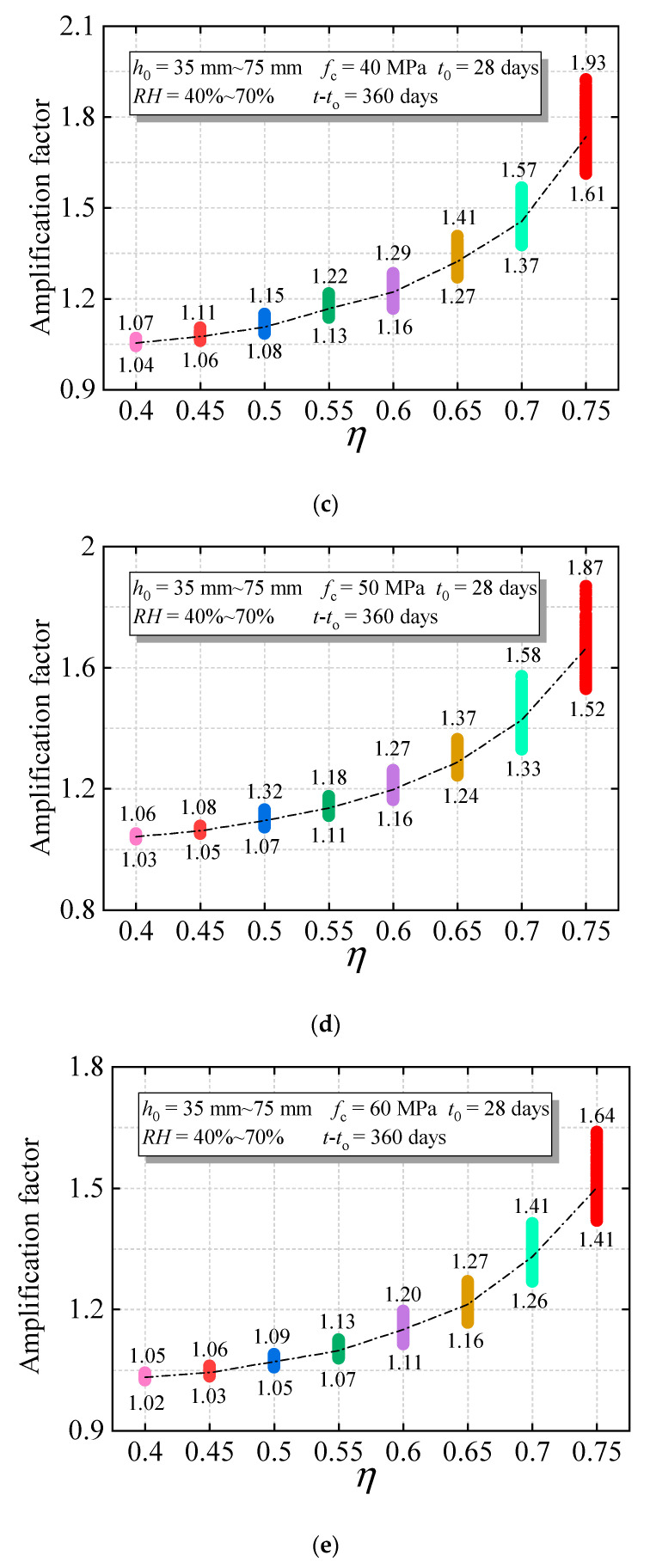
Confidence interval of nonlinear creep amplification coefficient: (**a**) amplification factor (20 MPa); (**b**) amplification factor (30 MPa); (**c**) amplification factor (40 MPa); (**d**) amplification factor (50 MPa); (**e**) amplification factor (60 MPa).

In order to study the variation of the concrete creep magnification factor, the relative humidity of the concrete is from 40% to 70%, the theoretical thickness of the component is from 35 mm to 75 mm, the duration of compression load is 360 days, and the concrete age at loading is 28 days. The standard value of the concrete strength is *f*_c_ = 20 MPa~60 MPa, and the calculation results of the creep amplification coefficient variation interval obtained by the calculation method in Section 3.2 are shown in Figure 6.

When the upper limit of the stress level interval is *η* = 0.75, and the creep deformation of the concrete component with a compressive strength of 20 MPa is 2.18 times higher than that of the linear calculation method, and considering the influence of the relative humidity and theoretical thickness of the component, the 95% confidence interval of the amplification coefficient is [1.91, 2.6], and the influence of nonlinear creep cannot be ignored. Under the same conditions, for the concrete members with strengths of 30 MPa~60 MPa, the range of the nonlinear amplification coefficient is [1.7, 2.11], [1.61, 1.93], [1.52, 1.87] and [1.41, 1.64], respectively. It can be seen that the amplification coefficient of nonlinear creep decreases with an increase in the concrete strength, mainly due to the decrease in the concrete damage. For the amplification coefficient under the stress level *η* = 0.4, it has a great influence on the concrete with 20 MPa, and the maximum creep strain increases by 18%, but it has little influence on the concrete with 30 MPa~60 MPa, and the maximum creep strain decreases from 8% to 3%, which is consistent with the existing research conclusions that the lower limit of the nonlinear creep stress level is calculated from 0.4, which is mainly related to the concrete strength.

For the concrete with the same strength, the creep amplification coefficient increases with the increase in the stress level, and the creep amplification coefficient changes nonlinearly. By comparing the nonlinear creep amplification factor of concrete with a compressive strength of 20 Mpa~60 Mpa, when the stress level rises from 0.4*f*_c_ to 0.6*f*_c_, the nonlinear increasing trend of the amplification coefficient is small, and, when the stress level is higher than 0.6*f*_c_, the nonlinear creep increases obviously, which is consistent with the research results by Mazzotti and Savoia [16]. The average statistics of the nonlinear amplification factor under different strengths and different stress levels are shown in Table 2.

**Figure 7:**
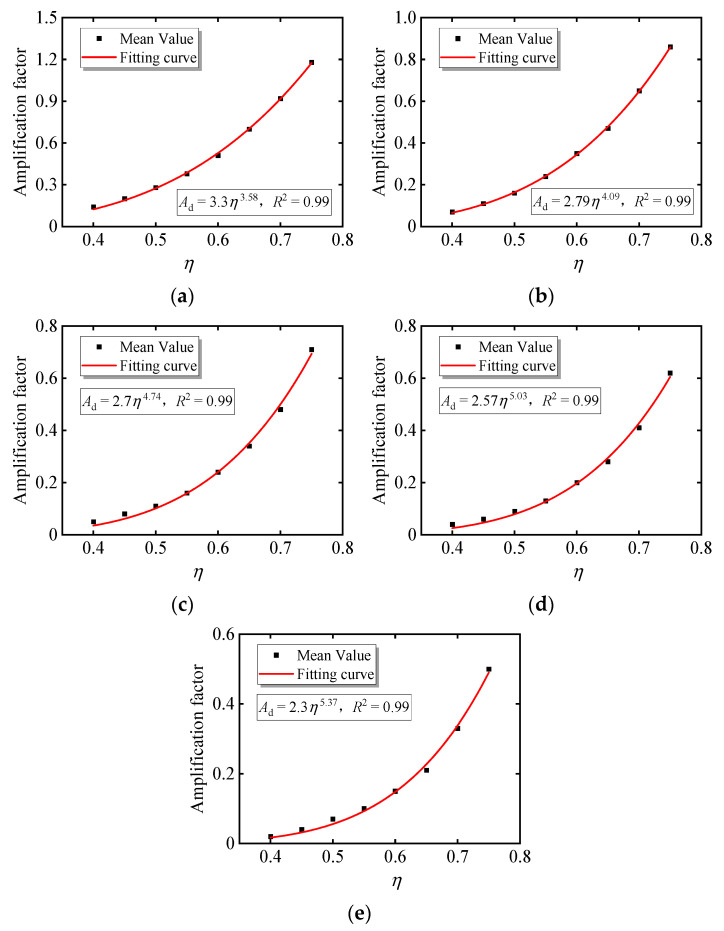
Curve fitting of mean value based on nonlinear amplification coefficient: (**a**) data fitting (20 MPa); (**b**) data fitting (30 MPa); (**c**) data fitting (40 MPa); (**d**) data fitting (50 MPa); (**e**) data fitting (60 MPa).

Nonlinear fitting is performed using the mean value of the creep magnification coefficient as a representative value, and the fitting function is in the form of a power function *y* = *ax^b^*, as shown in Figure 7. The fitting formulas of the standard values of compressive strengths from 20 MPa to 60 MPa are given. It can be seen from the figure that the coefficient formula varies with the change in the concrete strength, and the results are different from those in references [18,19,20,21,32]. The results in Figure 7 show that when considering the damage evolution, the nonlinear creep amplification coefficient is not only related to the stress level but also to the concrete strength.

**Figure 8:**
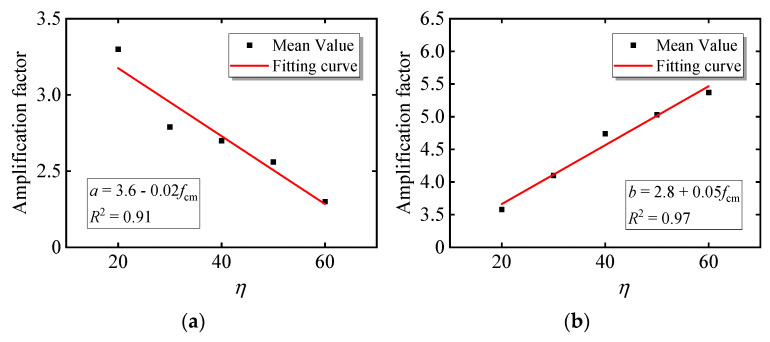
Power function correlation coefficient curve fitting: (**a**) coefficient *a*; (**b**) coefficient *b*.

The values of the power function coefficients of various concrete cylinder strengths *f*_c_ are shown in Table 3. It can be seen from the table that the coefficients ***a*** and ***b*** of the power function are related to the concrete axial strength when considering the concrete damage evolution. The coefficient *a* gradually decreases with the increase in the concrete axial strength. The coefficient *b* increases gradually with the increase in the concrete axial strength. Fit the correlation coefficients *a* and *b* with the strength values. From Figure 8, it can be seen that the correlation coefficient of the power function has a linear relationship with the standard value of the axial strength of the concrete, where the fitting coefficients are *a* = 3.6 − 0.02*f*_c_ and *b* = 2.8 + 0.05*f*_c_.

Therefore, considering the effects of the nonlinear creep of the concrete compression damage evolution, when the stress level ranges from 0.35 to 0.75, the creep amplification factor of the concrete damage evolution is:

(21) $$A_{v}=1+(3.6-0.02f_{c})\eta^{\left(2.8+0.05f_{c}\right)}$$

### 4.2. Comparative Analysis

**Figure 9:**
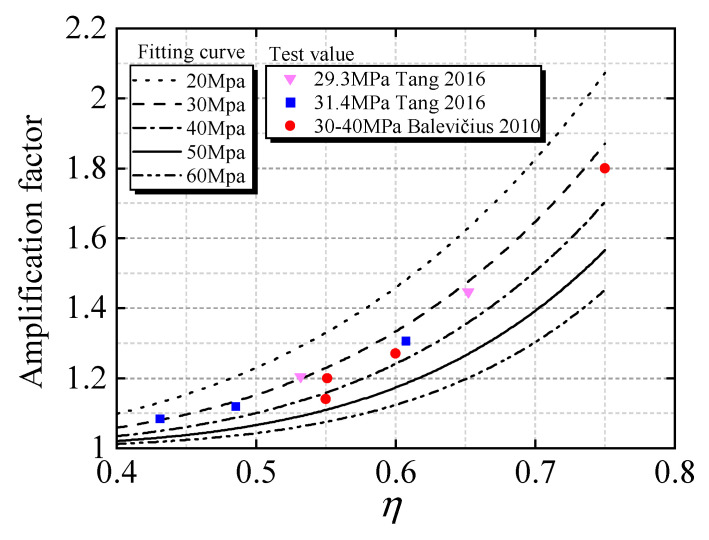
Comparative analysis of amplification factor compared with Tang [21] and Balevičius [42].

The development curve of the nonlinear creep amplification coefficient under varying concrete strengths is compared with the theoretical amplification coefficient provided in the literature [21,42], and the results are shown in Figure 9. For the amplification coefficient of the concrete with a compressive strength near 30 MPa~40 MPa, the fitting curve based on the mean value is in good agreement with the tested values.

**Figure 10:**
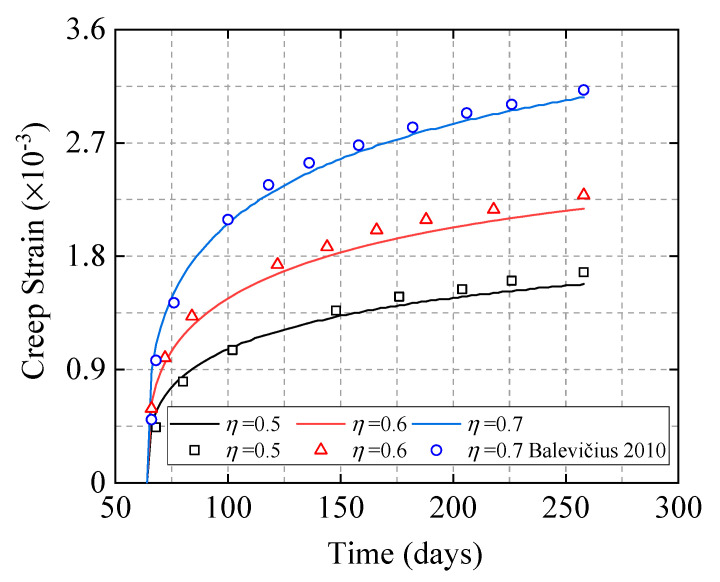
Creep strain test results [37] and formula predictions at gradient stress level of 0.5~07.

The nonlinear amplification coefficient is applied to the calculation of the concrete creep strain and compared with the strain value of the nonlinear creep test of the cylinder. Hamed [33] designed a set of nonlinear creep tests at 50% relative humidity and a holding time of 194 days, incorporating a gradient from 0.5 to 0.7 stress levels. It can be seen from Figure 10 that the calculated results are in good agreement with the experimental results, and the proposed calculation formula can better predict the nonlinear creep deformation of concrete with different stress levels.

Fu [43] compared the difference between the nonlinear creep prediction model and the measured value. The amplification coefficients of nonlinear creep proposed by different scholars [22] and codes [2,10] are selected as the reference group, and the results of the four nonlinear creep calculation results and test values are plotted in the same figure. The concrete strength is 46.9 MPa, and the stress level is divided into 0.61 and 0.64. The concrete age at loading is 28 days, and the remaining specific parameters [43] are shown in Table 4. Table 5 shows the nonlinear amplification factors of four models at the corresponding concrete strengths and stress levels.

**Figure 11:**
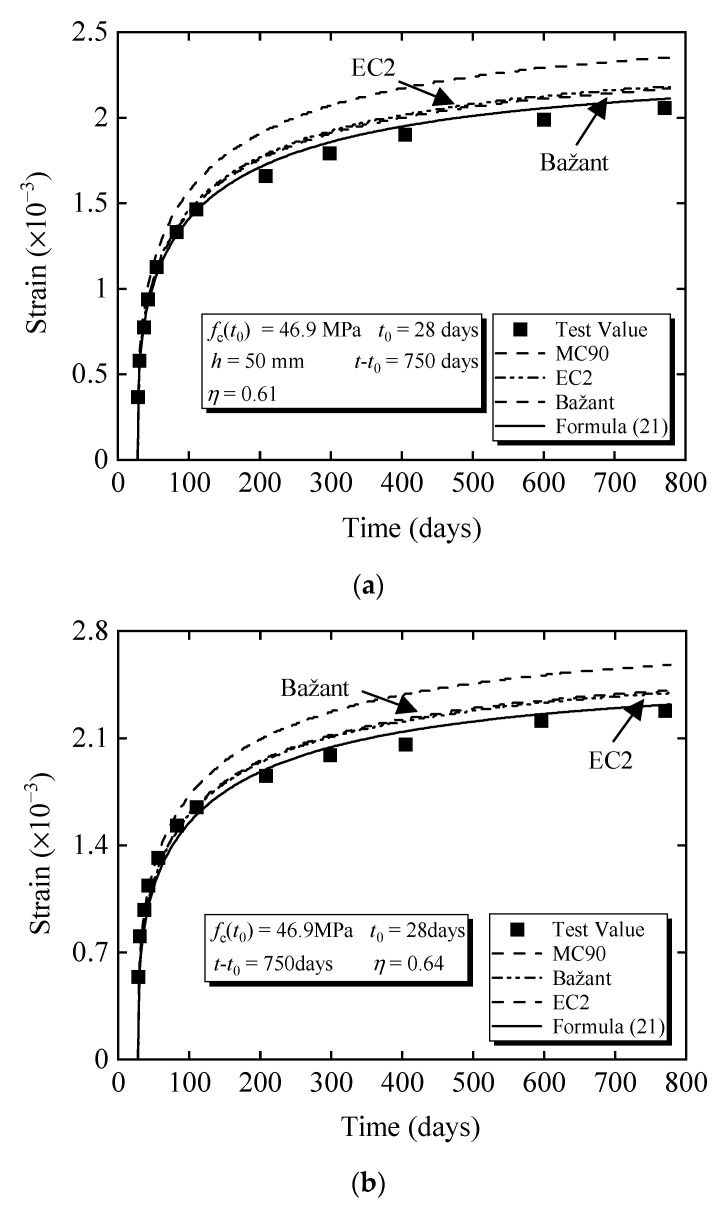
Comparative analysis of test strain: (**a**) strain comparison (*f*_c_ = 46.9 MPa and *η* = 0.61); (**b**) strain comparison (*f*_c_ = 46.9 MPa and *η* = 0.64).

It can be seen from Figure 11 that the EC2 and Bažant models are similar, but both are higher than the measured values. The calculation result of the MC90 model is the largest among all models, and the deviation is larger than that of the measured results. The calculation result of Formula (21) is the closest to the measured result, which shows that the calculation method of Formula (21) is effective.

## 5. Conclusions

It is believed that the creep strain calculated from the elastic stress–strain relationship of concrete is lower than concrete creep under higher compressive stress, mainly because the damage caused by concrete compression is ignored, including the development of interface cracking and internal mortar cracking. The following conclusions can be drawn:

(1) Considering the multi-level effects of concrete creep, it is recommended that the study of the nonlinear creep magnification factor in the medium stress state should take a stress level range from 0.35*f*_c_ to 0.75*f*_c_.
(2) The nonlinear creep model of concrete under different strengths is constructed by coupling the concrete damage evolution with elastic creep, which can better reflect the nonlinear creep of the concrete under a medium stress state and simplify the calculation process.
(3) Concrete nonlinear creep is related to stress level and concrete strength. The nonlinear creep amplification coefficient of concrete with the same strengths increases nonlinearly with an increase in the compressive stress level; when the concrete compressive stress level is determined, the nonlinear creep magnification factor gradually decreases significantly with an increase in the concrete strength. The nonlinear creep amplification coefficient is positively related to the stress level and negatively related to the concrete compressive strength.

## Figures and Tables

**Table 1:** Mechanical properties of concrete.

Concrete Age (Days)	Compressive Strength (MPa)		Modulus of Elasticity (GPa)
	Cubic	Prism	
7	59.0	49.0	42.1
14	67.0	52.8	44.1
28	79.6	67.0	46.6
45	84.5	71.2	47.7
90	88.6	74.0	47.8

**Table 2:** Mean value of creep amplification coefficient.

η	Concrete Compressive Strength *f*_c_ (MPa)				
	20	30	40	50	60
0.40	1.14	1.07	1.05	1.04	1.03
0.45	1.21	1.11	1.07	1.06	1.04
0.50	1.31	1.16	1.09	1.09	1.07
0.55	1.36	1.24	1.16	1.13	1.10
0.60	1.51	1.35	1.22	1.18	1.15
0.65	1.73	1.47	1.32	1.28	1.21
0.70	1.92	1.65	1.45	1.41	1.33
0.75	2.18	1.86	1.73	1.66	1.5

**Table 3:** Power function coefficient value.

Power function Coefficient	Concrete Compressive Strength *f*_c_ (MPa)				
	20	30	40	50	60
*a*	3.3	2.79	2.7	2.56	2.3
*b*	3.58	4.10	4.74	5.03	5.37

**Table 4:** Test parameters of cylinder components.

Specimen	*D*	*h*_0_	*RH*	*σ*(*t*_0_)	*E*_c_(*t*_0_)	η
Number	(mm)	(mm)	(%)	(MPa)	(MPa)	
1	100	50	65	46.9	3.5 × 10^4^	0.61
2	100	50	65	46.9	3.5 × 10^4^	0.64

**Table 5:** Nonlinear amplification coefficient proposed by scholars.

Concrete Strength	η	Bažant [22]	MC90 [10]	EC2 [2]	Formula (21)
46.9 MPa	0.61	1.26	1.37	1.27	1.23
46.9 MPa	0.64	1.34	1.43	1.33	1.28

## Data Availability

Not applicable.
